# Altered Theca and Cumulus Oocyte Complex Gene Expression, Follicular Arrest and Reduced Fertility in Cows with Dominant Follicle Follicular Fluid Androgen Excess

**DOI:** 10.1371/journal.pone.0110683

**Published:** 2014-10-16

**Authors:** Adam F. Summers, William E. Pohlmeier, Kevin M. Sargent, Brizett D. Cole, Rebecca J. Vinton, Scott G. Kurz, Renee M. McFee, Robert A. Cushman, Andrea S. Cupp, Jennifer R. Wood

**Affiliations:** 1 Department of Animal Science, University of Nebraska-Lincoln, Lincoln, Nebraska, United States of America; 2 USDA-ARS Roman L. Hruska U.S. Meat Animal Research Center, Clay Center, Nebraska, United States of America; USA, United States of America

## Abstract

Aspiration of bovine follicles 12–36 hours after induced corpus luteum lysis serendipitously identified two populations of cows, one with High androstenedione (A4; >40 ng/ml; mean = 102) and another with Low A4 (<20 ng/ml; mean = 9) in follicular fluid. We hypothesized that the steroid excess in follicular fluid of dominant follicles in High A4 cows would result in reduced fertility through altered follicle development and oocyte maternal RNA abundance. To test this hypothesis, estrous cycles of cows were synchronized and ovariectomy was performed 36 hours later. HPLC MS/MS analysis of follicular fluid showed increased dehydroepiandrosterone (6-fold), A4 (158-fold) and testosterone (31-fold) in the dominant follicle of High A4 cows. However, estrone (3-fold) and estradiol (2-fold) concentrations were only slightly elevated, suggesting a possible inefficiency in androgen to estrogen conversion in High A4 cows. Theca cell mRNA expression of *LHCGR, GATA6*, *CYP11A1,* and *CYP17A1* was greater in High A4 cows. Furthermore, abundance of *ZAR1* was decreased 10-fold in cumulus oocyte complexes from High A4 cows, whereas *NLRP5* abundance tended to be 19.8-fold greater (*P* = 0.07). There was a tendency for reduction in stage 4 follicles in ovarian cortex samples from High A4 cows suggesting that progression to antral stages were impaired. High A4 cows tended (P<0.07) to have a 17% reduction in calving rate compared with Low A4 cows suggesting reduced fertility in the High A4 population. These data suggest that the dominant follicle environment of High A4 cows including reduced estrogen conversion and androgen excess contributes to infertility in part through altered follicular and oocyte development.

## Introduction

Ovarian steroidogenesis is regulated in a spatial and temporal manner within the granulosa and theca cells of the follicle. This regulation is achieved by cell-specific expression of steroidogenic enzymes which regulate conversion of cholesterol to steroid hormones [1]. In theca cells, cholesterol is transported from the cytosol into the mitochondria by the steroidogenic acute regulatory protein (STAR) [2], [3] where it is converted to pregnenolone by cytochrome P450 side-chain cleavage (P450scc or CYP11A1) enzyme. In species that utilize the Δ5 steroidogenic pathway, including bovine and human, pregnenolone is converted first to 17α-hydroxypregnenolone and then to dehydroepiandrosterone (DHEA) via cytochrome P450 17α-hydroxylase (P450c17 or CYP17A1). The third carbon of DHEA is subsequently dehydrogenated by 3β-hydroxysteroid dehydrogenase (HSD3B) to form androstenedione (A4) [1]. Cytochrome P450-aromatase (P450arom or CYP19A1) hydrolyzes A4 to form estrone which is then modified by the enzyme 17β-hydroxysteroid dehydrogenase (HSD17B) to form estradiol (E2). Conversely, A4 can first be converted to testosterone (T) by HSD17B and then hydroxylated via CYP19A1 to form E2 [1].

The expression and activity of steroidogenic enzymes in the theca and granulosa cells is primarily regulated by the gonadotropins, luteinizing hormone (LH) and follicle stimulating hormone (FSH) [4]. Pulsatile release of LH from the anterior pituitary binds to LH receptors on theca cells and activates cAMP-dependent signaling which increases the activity and expression of STAR, CYP11A1, HSD3B, and CYP17A1 [1] [5]. Likewise, FSH activates cAMP-dependent signaling in granulosa cells resulting in increased activity and expression of CYP19A1 [6]. Ovarian growth factors including bone morphogenetic proteins and insulin-like growth factor also modify basal and gonadotropin-dependent steroid synthesis indicating important local control of theca and granulosa cell steroidogenesis [7], [8], [9]. Increased expression of steroidogenic enzyme genes by gonadotropins and local growth factors is mediated by several transcription factors. For example, the regulation of *STAR, CYP11A1, CYP17A1*, and *HSD3B* by NR5A1 (steroidogenic factor 1) and CCAAT/enhancer binding proteins has been well documented [10], [11]. Other studies [12], [13] show that the transcription factor GATA6 also regulates the expression of steroidogenic enzyme genes in granulosa and theca cells, respectively.

Competence of the oocyte for fertilization and embryonic development has been correlated to several molecular and phenotypic characteristics including oocyte diameter, density and distribution of organelles, and accumulation of RNA and proteins [14]. Alterations in the local ovarian environment, which includes the milieu of steroid hormones and growth factors produced by the oocyte and somatic cells of the follicle, impact these characteristics and thereby reduce the quality of the ovulated oocyte. For example, increased lipids induce endoplasmic reticulum stress and mitochondrial dysfunction [15], [16] while androgen excess has been associated with altered oocyte mRNA abundance [17]. Recent studies have emphasized the importance of maternal RNAs retained in the ovulated oocyte for successful embryonic development. Specifically, these maternal effect gene transcripts are required for fertilization of the oocyte, formation of the zygote, and/or initiation of cell cleavage prior to activation of embryonic genome [18]. Several reports, using a candidate gene approach in rodents have validated the importance of maternal effect genes with alterations in DNA methylation and termination of early embryogenesis reported in knockout models [19], [20], [21], [22].

Previous reports highlight the importance domesticated livestock species, specifically cows, may have in increasing our understanding of reproductive technologies and reproductive disorders in humans [23], [24], [25]. Similar to humans, cows are monovulatory and can present similar pathological conditions including luteinization of anovulatory follicles and stress related suppression of follicle growth and ovulation [24]. In addition, crucial ovarian tissues including granulosa cells which have not been exposed to an LH surge, theca cells, and cumulus-oocyte complexes are readily available for studies. Interestingly, in our cow herd, we have identified a population which exhibits increased intrafollicular androgens. Thus, the objective of the current study was to determine how altered follicular environment in cows with High A4 contributes to reduced fertility by evaluating follicular progression and mRNA abundance in somatic cells and cumulus-oocyte-complexes.

## Materials and Methods

### Animals

Beef cows from the physiology herd located at the University of Nebraska Agricultural Research and Development Center (ARDC) were used in these studies. The physiology herd consists of approximately 230 composite beef cows [75% Red Angus×25% MARC III (¼ Red Angus, ¼ Hereford, ¼ Pinzgauer, ¼ Red Poll)]. The University of Nebraska-Lincoln Institutional Animal Care and Use Committee approved all procedures and facilities used in this experiment.

### Ovarian aspirations

Twenty-six cows were randomly selected from the physiology herd and estrous cycles synchronized by lysing the CL with two injections of PGF_2α_ (PG; 25 mg/mL; i.m.; Lutalyse, Pfizer Animal Health, New York, NY) 14 days apart (Figure S1A). This protocol naturally stimulates progesterone removal with CL lysis, and an ovulatory surge of LH occurs from 56 to 72 hours after the second injection of PG. This study was conducted on these cows over multiple estrous cycles and years. An epidural was given with 2% lidocaine (5 mL/cow) and follicle aspirations were conducted at 12, 18, 24, 30, or 36 hours after administration of the second injection of PG. An ultrasound system (ALOKA SSD-500 V, ALOKA Co. Ltd., Tokyo, Japan) with a 5 MHz transvaginal convex human transducer (ALOKA UST-981-5) adapted for the bovine with attached stainless steel needle guide was used to perform transvaginal, ultrasound-guided aspiration of dominant follicles with an 18-gauge needle. The follicular fluid was aspirated and separated from granulosa cells by centrifugation. Follicular fluid was stored at –80°C until further analysis.

### Ovariectomies

To allow for theca cell collection and increase recovery rate of cumulus oocyte complexes, ovariectomies were conducted. Cows were randomly selected from the herd and estrous cycles synchronized with a protocol that utilizes GnRH and a controlled internal drug release device (CIDR; 1.38 g progesterone, Zoetis, Florham Park, NJ) (Fig. S1B). Ovariectomy (n = 64) was performed approximately 36 hours after CIDR removal, which coincided with administration of PG to eliminate a functional CL. This estrous synchronization protocol allows for better synchronization of follicular waves and thus, more follicles are retrieved from cows at similar stages of follicular development.

Prior to surgery, transrectal ultrasonography (ALOKA SSD-500 V and UST-5821-7.5, ALOKA Co. Ltd., Tokyo, Japan) was performed by a single technician to evaluate the presence and size of any large follicles or CLs. Both ovaries were removed via a right flank laparotomy using aseptic techniques similar to a previously reported procedure [26].

### Ovarian tissue collection

Ovaries were weighed, ovarian length and width recorded, and surface antral follicle count was performed. The dimensions of the largest (dominant) and second largest (subordinate) follicles were also measured and recorded. Follicular fluid was aspirated from these follicles, individual cumulus oocyte complexes retrieved, and theca cells collected via microdissection. To determine purity of theca cell and granulosa cell samples quantitative RT-PCR (QPCR) was performed to determine mRNA abundance of theca and granulosa specific markers. Gene expression for aromatase (*CYP19A1*), isolated in granulosa cells, was not detected in theca cells. Similarly, mRNA abundance of *CYP17A1*, located in theca cells, was not detected in granulosa cells. Granulosa cells were collected from the follicular fluid as described above. Granulosa and theca cell weights were recorded and granulosa cell volumes estimated. The ratio of theca to granulosa cells was also determined by dividing theca cell weight (g) by granulosa cell weight (g). The volume of follicular fluid aspirated from each follicle was measured and then stored at –80°C. Both granulosa and theca cells were homogenized in Tri-reagent (Sigma-Aldrich, St. Louis, MO) for RNA isolation.

### Hormone assays

Follicular fluid 17β-estradiol (E2) and progesterone (P4) concentrations were determined by radioimmunoassay (RIA). Estradiol RIA was carried out as previously described [27]. Intra- and inter-assay coefficients of variation were 14.4% and 22.8%, respectively. Progesterone RIA was carried out using the Coat-a-Count assay kit (Diagnostic Products Corporation, Los Angeles, CA). Intra- and inter-assay coefficients of variation for P4 were 1.6% and 5.2%, respectively. Follicular fluid A4 and DHEA concentrations were determined utilizing a human A4 ELISA kit (Alpha Diagnostics International, San Antonio, TX) and DHEA ELISA kit (Fitzgerald Industries International, Acton, MA), respectively. The intra- and inter-assay coefficients of variation for A4 were 6.5% and 5.7%, respectively. The intra- and inter-assay coefficients of variation for DHEA were 2.2% and 5.9%, respectively. Follicles were classified as estrogen active if the ratio of E2/P4 was greater than 1. Cows were classified as High or Low A4 based on dominant follicle A4 concentration for both follicle aspiration (n = 15 High A4, n = 11 Low A4) and ovariectomy (n = 36 High A4, n = 28 Low A4) procedures, respectively.

### High performance liquid chromatography-tandem mass spectrometry (HPLC-MS/MS)

To simultaneously analyze the concentration of multiple steroid hormones from a single sample, follicular fluid was analyzed using HPLC-MS/MS. Samples were selected based on ELISA A4 concentration with High A4 (n = 7) samples having the greatest concentration and Low A4 (n = 7) samples having the lowest concentration of A4, respectively. Samples were analyzed by Biocrates Life Sciences AG (Innsbruck, Austria) using the SterolDQ HPLC-MS/MS kit as previously reported [28].

### RNA extraction and RT

Total RNA was extracted from each cumulus oocyte complex (COC) utilizing the Ambion MicroPoly (A) Purist kit (Life Technologies Corp., Carlsbad, CA). After RNA extraction, linear amplification (1 round) and conversion to cDNA was completed utilizing WT Ovation RNA amplification system (NuGen Technologies Inc., San Carlos, CA). Previous studies have demonstrated that this amplification system reproducibly increases the total amount of RNA in a linear fashion without amplification artifacts or bias [29], [30]. Total RNA was extracted from granulosa and theca cells using Tri Reagent (Sigma-Aldrich). The extracted RNA was subsequently converted to cDNA using SuperScript III and random primers (Invitrogen, Carlsbad, CA) following standard procedures previously reported [31].

### Quantitative RT-PCR

Quantitative RT-PCR was performed to evaluate mRNA abundance in theca cells and COCs as previously reported [31], [32]. Primers and probes for target mRNAs were designed using Primer Express 1.5 (Applied Biosystems, Foster City, CA; Sup. Table 1) and synthesized (Integrated DNA Technologies Inc., Coralville, IA). Samples were run in triplicate in separate consecutive wells and replicates with SD greater than 0.3 were removed from the analysis. Primers were also designed for the constitutively expressed mRNAs, glyceraldehyde-3-phosphate dehydrogenase (*GAPDH*), ribosomal protein L 15 (*RPL-15*), and ribosomal protein L 19 (*RPL-19*) (Sup. Table 1). The stability of the constitutively expressed mRNAs was calculated using Normfinder [33], [34] and based on this analysis, candidate gene mRNA abundance was normalized using the geometric mean of *GAPDH* and *RPL-15*. The resulting normalized data for each candidate mRNA was then compared to the mean normalized mRNA abundance in Low A4 samples and expressed as a fold change.

**Table 1:** Ovarian and follicle phenotypic measurements for Low A4 and High A4 cows.

Item	Low A4^1^	High A4^2^	SEM	*P*-value
Follicle aspirations^3^				
n	11	15		
Follicle diameter, mm	13.38	13.27	1.18	0.90
Follicular fluid, mL	1.57	0.98	0.41	0.08
Ovariectomies^4^				
n	28	36		
Follicle diameter, mm	14.21	15.01	0.75	0.30
Follicular fluid, mL	1.09	1.33	0.10	0.07
Theca cell weight, g	0.07	0.08	0.01	0.23
Granulosa cell weight, g	0.08	0.06	0.01	0.24
Theca:Granulosa weight	1.20	1.83	0.30	0.05
Ovarian area, mm^2^	629	684	46	0.25
Ovarian weight, g	7.27	8.13	0.72	0.29
Surface AFC^5^	53	46	4	0.19
Ultrasound AFC^5^	28	31	2	0.26
Ovarian area, mm^2^	629	684	46	0.25

1 Dominant follicle follicular fluid A4 concentration of <20 ng/mL.

2 Dominant follicle follicular fluid A4 concentration of >40 ng/mL.

3 Follicle aspirations were taken 12–48 hours after two injections of Prostaglandin F2alpha (PG).

4 Ovariectomies were conducted after modified cosynch protocol 36 hours after PG.

5 Where AFC = antral follicle count.

### Ovarian histology

After dominant follicle removal, ovarian cortex samples were collected to determine if there were differences in folliculogenesis within the ovary based on cow A4 classification as quantitated by follicles at particular stages of development within the cortex. Tissues were fixed in Bouin’s and embedded in paraffin. Samples were sectioned (5 µm) and 3 serial sections placed on each slide. Sections from slides one, five, and ten were selected from each individual, stained with hematoxylin and eosin, imaged, and follicles classified in one of five groups: 0) primordial follicle- an oocyte surround by a single layer of squamous pregranulosa cells; 1) transitional follicle or early primary- an oocyte surrounded by mostly squamous pregranulosa cells with some cuboidal granulosa cells; 2) primary follicle- an oocyte surround by 1–1.5 layers of cuboidal granulosa cells; 3) secondary follicle- an oocyte surrounded by 2 or more cuboidal granulosa cells; 4) follicle no larger than 1 mm in diameter with an oocyte surrounded by 2 or more layers of granulosa cells that contains a distinct antrum [35], [36]. Follicle staging and counting was conducted by two individuals and averaged to determine the number of follicles at each stage present.

### Statistical analyses

Follicle aspirations were performed over a 2-year period. Data were analyzed utilizing PROC GLIMMIX of SAS (SAS Institute Inc., Cary, NC) with the initial model including classification (High vs. Low A4), time, and the interaction. There was no classification × time (*P*>0.15) interaction; thus, it was removed from the model and the main effects of A4 classification and time analyzed for hormone concentrations and follicle characteristics.

Ovariectomies were performed over a 5-year period with approximately 10–14 cows ovariectomized each replication. Thus, each surgery was considered a replication and animals considered the experimental unit. Ovarian phenotypic measurements, including surface and ultrasound derived AFC, ovary size and weight, theca cell weight, follicle size and follicular fluid volume were analyzed utilizing the MIXED procedure of SAS (SAS Institute Inc., Cary, NC). Furthermore, hormone concentrations and gene expression in both theca and COC were analyzed using the MIXED procedure of SAS. The model included A4 classification (High or Low) as the main effect with replication as the random effect. The original model included A4 classification and age as fixed effects with replication as a random effect. Age was not different among treatments, and thus was removed from the model. Data for Maternal antigen embryos require (*NLRP5)*, zygote arrest-I (*ZAR1*), eukaryotic translation initiation factor 2C (*EIF2C2)*, endoribonuclease (*DICER)*, developmental pluripotency associated protein 3 (*DPPA3*) in the COC and 17α-hydroxylase/17,20 lyase (*CYP17A1*), cholesterol side chain cleavage enzyme (*CYP11A1)*, *GATA6,* luteinizing hormone/choriogonadotropin receptor (*LHCGR*) in the theca cells were log transformed to meet normal distribution assumptions. A *P*-value≤0.05 was considered significant.

## Results

### Follicle aspirations indicated divergent populations of cows based on A4 concentrations in the follicular fluid of the dominant follicle

**Figure 1:**
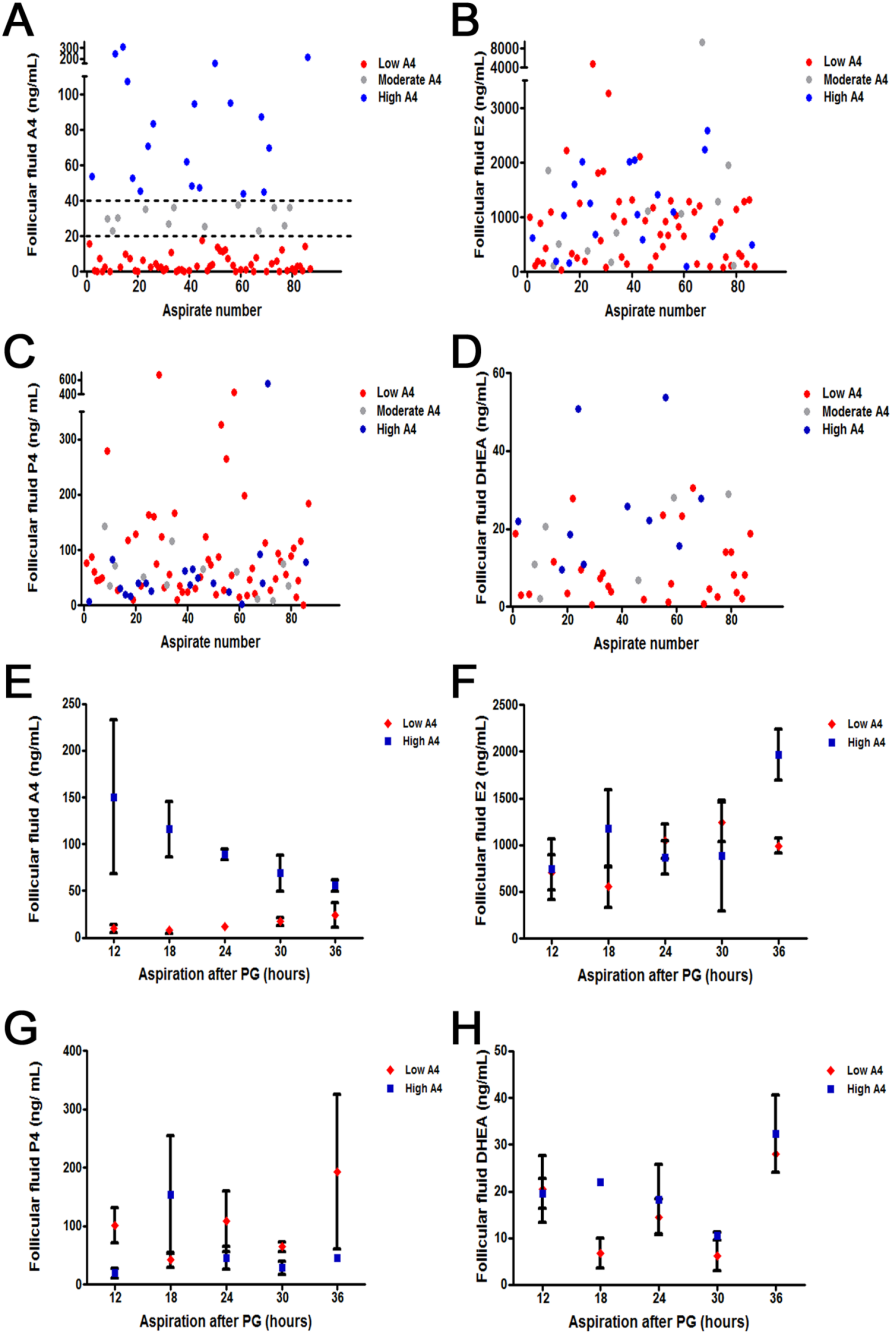
Individual Aspirate (A–D) and Temporal (E–H) differences in Dominant Follicle Steroid Hormone Concentrations Following PG. Androstenedione (A4) concentrations (A) naturally separate above (blue) or below (red) the average (43 ng/mL) with some samples indicated in the moderate region (20–40 ng/mL; grey). Estradiol (E2; B), Progesterone (P4; C) and dehydroepiandrosterone (DHEA; D) concentrations are similar regardless of A4 concentrations. At each time point measured (12–36 hours) androstenedione concentrations (E) are increased in High A4 cows; although E2 concentration (F), P4 concentration (G), and DHEA concentration (H) were similar for High and Low A4 cows.

To assess follicular fluid steroid profiles of preovulatory stage follicles, PGF_2α_ (PG) was administrated and follicles aspirated 12–36 hours later. In each aspirate, E2, and P4 concentrations were measured and follicles were subsequently classified as either E2-active (E2:P4>1) or E2-inactive (E2:P4≤1) [37]. Surprisingly, when the A4 concentrations of fluid from E2-active follicles were measured, 64% of E2-active aspirates had A4 levels less than 20 ng/mL. However, 22% had A4 levels greater than 40 ng/mL which was two standard deviations greater than the mean A4 concentration of Low A4 cows. (Fig. 1A). Cows were classified in the same A4 group 76% (*P* = 0.005) of the time. Furthermore, if follicles were aspirated ≥24 hours after PG administration, cows remained in the same A4 classification group 80% (*P* = 0.07) of the time. This is likely due to the fact that overall averages of androstenedione in our herd were greater prior to 24 hours PG in developing follicles. Obtaining follicles 24 hours after PG also ensured that these follicles were estrogen active and efficiently converting androgens to estrogens. Despite the differences in A4 concentration, aspirates did not segregate based on follicular fluid E2 (Fig. 1B), P4 (Fig. 1C), or DHEA (Fig. 1D) concentrations.

Concentrations of A4, E2, P4, and DHEA in the follicular fluid at each individual time point were also compared between High A4 and Low A4 cow populations. Follicular fluid hormone concentrations were averaged within each A4 classification group and mean concentration displayed (Fig. 1E–G). Aspirations were completed on random cows within the herd at differing time points resulting in some time points missing High or Low A4 data points (Fig. 1E–H). Androstenedione follicular fluid concentration was greater in dominant follicles aspirated from High vs. Low A4 classified cows (Fig. 1E; *P*≤0.04) at each time point. Likewise, DHEA (Fig. 1H) concentration tended (*P* = 0.07) to be greater in the High A4 group but only 18 hours after induction of ovulation with PG. Conversely, E2 (Fig. 1F) and P4 (Fig. 1G) concentrations were not different between High and Low A4 cows at any time point.

Ultrasound of the dominant follicles at the time of aspiration revealed similar (*P* = 0.90) follicle diameters from High and Low A4 cows (13.27 vs. 13.38±1.18 mm; Table 1). However, follicular fluid volume tended (*P* = 0.07) to be greater for Low vs. High A4 cows (1.57 vs. 0.98±0.41 mL).

### Follicular fluid hormone concentrations differ in High and Low A4 CIDR-synchronized cows ovariectomized 36 hours after PGF2a

Based on these altered hormone concentrations and the natural segregation of populations within the herd, we hypothesized that the steroid excess in follicular fluid of dominant follicles in High A4 cows would result in reduced fertility through altered follicle development and oocyte maternal RNA abundance. To test this hypothesis, the first wave of follicle development was synchronized in cows utilizing GnRH and exogenous progesterone and ovariectomies performed 36 hours after PG administration.

Similar to the aspiration data, A4 concentration in the follicular fluid of E2-active dominant follicles collected from ovariectomized cows differed substantially when cows were classified as High or Low A4 (Table 2) via RIA or ELISA. As expected, A4 concentration was approximately 24.5-times greater (*P*<0.0001) in the follicular fluid of High A4 cows compared with Low A4 cows. There was no difference (*P* = 0.16) in P4 concentration based on cow A4 classification; however, dehydroepiandrosterone (DHEA) concentration was 2-fold greater (*P* = 0.01) in the follicular fluid of High A4 compared with Low A4 cows. Estradiol concentration in the follicular fluid was also increased in High A4 compared with Low A4 cows but only 1.5-fold (*P* = 0.001). Despite these differences in follicular fluid steroid concentrations, no differences in circulating plasma levels of A4 were detected between High and Low A4 cows (Table 2).

**Table 2:** Follicular fluid and Plasma Hormone concentrations for Low A4 and High A4 cows.

Item	Low A4^1^	High A4^2^	SEM	*P*-value
Follicular Fluid Steroid data RIA/ELISA				
n	28	36		
DHEA, ng/mL	11	22	4	0.01
Androstenedione, ng/mL	6	155	24	<0.0001
Estradiol, ng/mL	1735	2685	274	0.001
Progesterone, ng/mL	225	146	74	0.16
Plasma Steroid Hormone data RIA/ELISA				
n	14	17		
Androstenedione, pg/mL	83	72	7	0.23
n	28	36		
AMH, ng/ml	.1344	.1299	28	0.91

1 Dominant follicle follicular fluid A4 concentration of <20 ng/mL.

2 Dominant follicle follicular fluid A4 concentration of >40 ng/mL.

**Figure 2:**
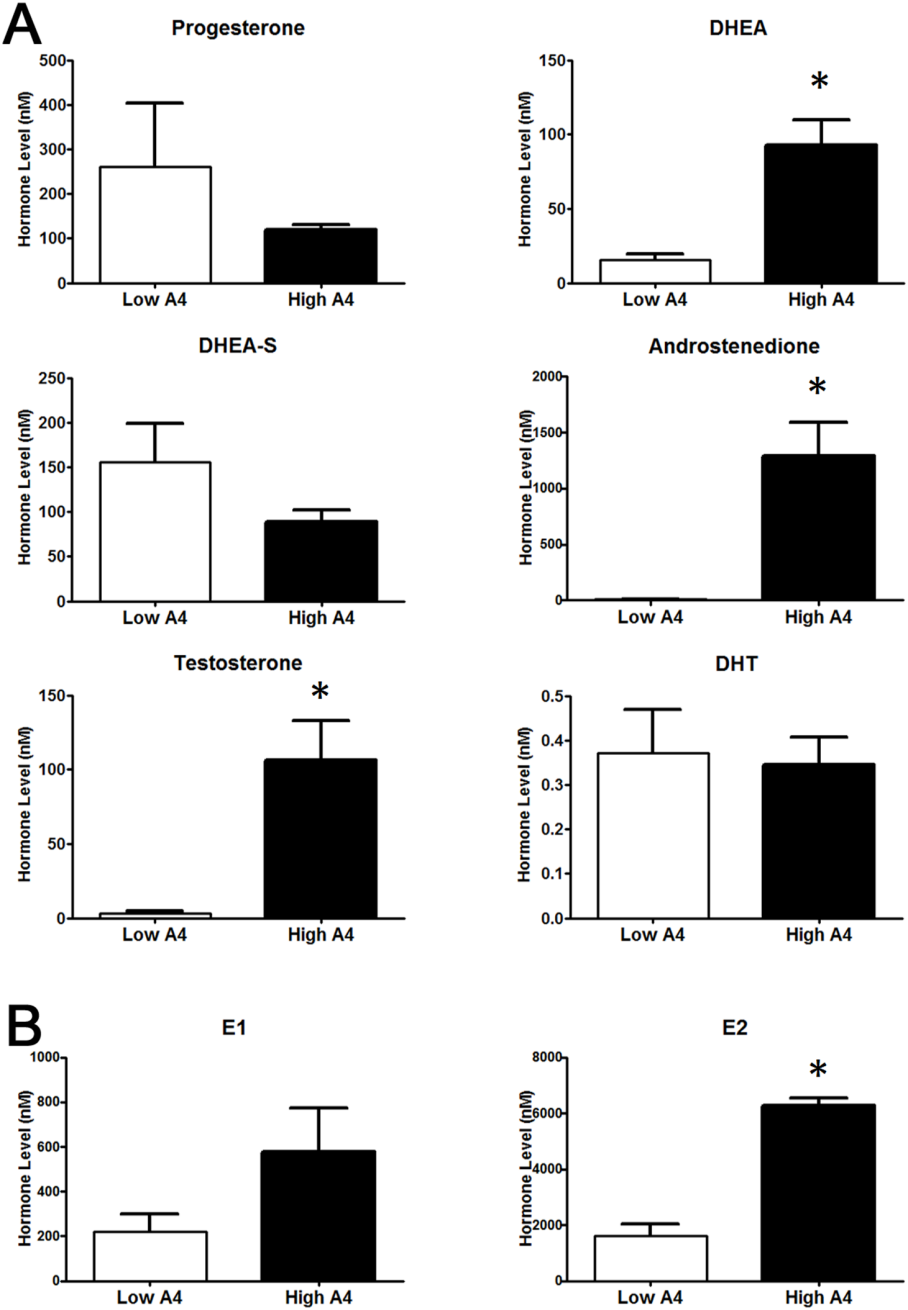
HPLC/MS-MS Analysis of Dominant Follicle Sex Steroid Concentrations Collected after Ovariectomy. Differences in concentration of androgens (A) and estrogens (B) was determined by HPLC/MS-MS (Biocrates Life Sciences AG) analysis for High A4 (black bars) and Low (white bars) A4 cows. *, *P*≤0.05.

In order to assess the relative concentrations of multiple steroids in a single follicular fluid sample, fluid collected from the E2-active dominant follicles of 7 High A4 (mean A4 = 1293.0±296.6 nM) and 7 Low A4 (mean A4 = 8.2±1.1 nM) cows were subjected to HPLC/MS/MS. Testosterone (T) concentration (Fig. 2A) was increased 30.8-fold (*P* = 0.002) in High A4 samples; whereas, estrone (E1) concentrations tended to be increased 2.64-fold (P  = 0.11, Fig. 2B). Similar to the individual hormone assays carried out on follicular fluid from all cows in the ovariectomy trial, DHEA, A4, and E2 (Table 1, Fig. 2A and B) concentrations were also increased (*P*≤0.01) in High A4 samples. Interestingly, when calculating the conversion rates of androgens and estrogens in the follicular fluid, High A4 cows had a 7-fold increase in A4:DHEA (*P* = 0.0005) compared with Low A4 cows. However the ratio of A4 to T (T:A4) was reduced in High A4 cows (*P* = 0.05). Similarly High A4 cows had reduced E1:A4 (*P* = 0.01) and E2:T (*P* = 0.04) compared with Low A4 cows; suggesting that there was less efficient conversion of androgens to estrogens in dominant follicles of High A4 cows.

**Figure 3:**
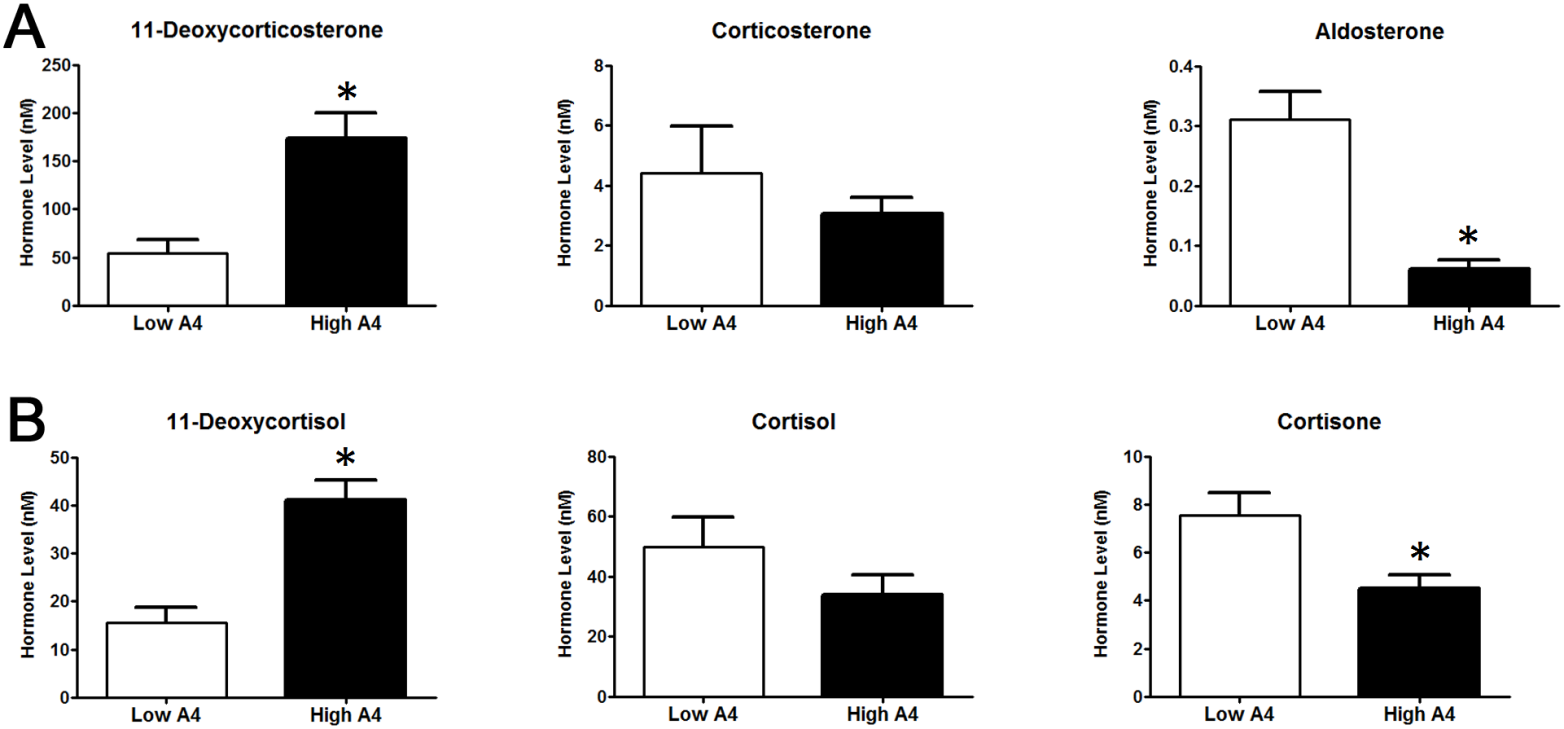
HPLC/MS-MS Analysis of Dominant Follicle Mineralocorticoid and Glucocorticoid Concentrations Collected after Ovariectomy. Difference in mineralocorticoid (A) and glucocorticoid (B) concentration in dominant follicle determined by HPLC/MS-MS (Biocrates Life Sciences AG) analysis for High A4 (black bars) and Low (white bars) A4 cows.*P≤0.05.

Interestingly, precursors to aldosterone (11-deoxycorticosterone; Fig. 3A) and cortisone (11-deoxycortisol; Fig. 3B) were elevated in follicular fluid from High A4 dominant follicles but these did not result in elevated aldosterone and cortisone since these hormones were reduced in follicular fluid from High A4 cows.

### Steroidogenic enzyme gene expression is increased in theca cells of High A4 cows

**Figure 4:**
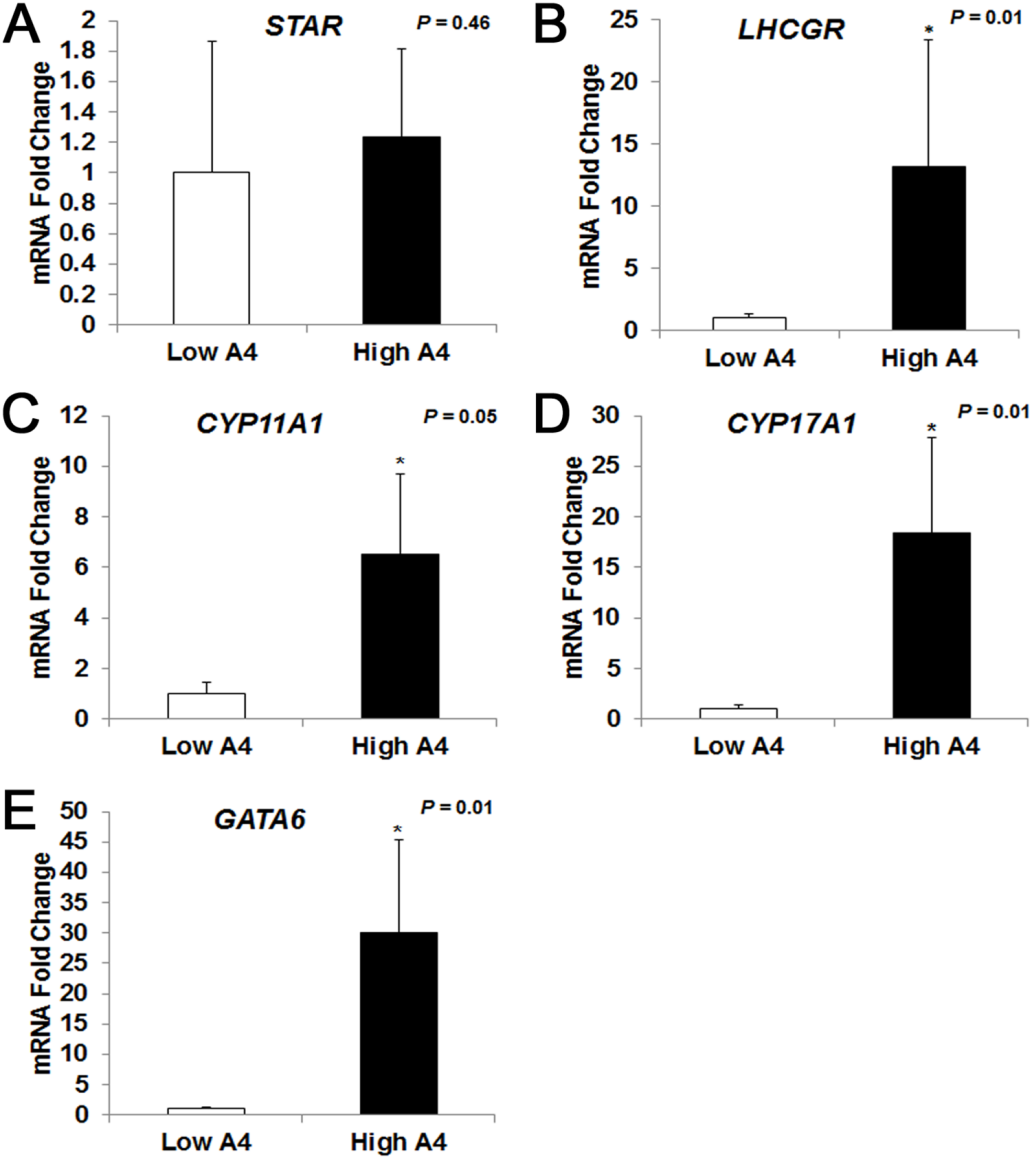
High A4 Cows Have Increased Steroidogenic Gene Expression in Theca Cells. Quantitative RT-PCR results for *StAR* (A), *LHCGR* (B), *CYP11A1* (C), *CYP17A1* (*D*), and *GATA6* (E) in theca cells collected from dominant follicles of High A4 (black bars, n≥12) and Low A4 (white bars, n≥19) cows. The geometric mean of *GAPDH* and *RPL-15* was used as an endogenous control. Data for *CYP11A1*, *CYP17A1*, *LHCRG*, and *GATA6* were log transformed to meet normal distribution assumptions. Graphs represent a fold change in mRNA abundance with Low A4 set as control (1). The mean ± SEM normalized values are presented. *, *P*≤0.05.

Theca and granulosa cells are important regulators of steroid synthesis in the ovary. Therefore, the mRNA abundance of steroidogenic enzymes was analyzed. In theca cells, the mRNA abundance of *StAR*, which traffics cholesterol from the outer to inner mitochondrial membrane was not different (*P* = 0.46, Fig. 4A) between High A4 and Low A4 cows. However, the abundance of *CYP11A1* mRNA, which is responsible for the conversion of cholesterol to pregnenolone, was 6.5-fold greater (*P* = 0.05, Fig. 4C) in High A4 compared with Low A4 theca cells. Furthermore, *CYP17A1* which converts pregnenolone to 17-OH pregnenolone and ultimately dehydroepiandrosterone (DHEA), exhibited an 18.4-fold increase (*P* = 0.01, Fig. 4D) in mRNA abundance in High vs. Low A4 theca cells. Luteinizing hormone directly increases expression of steroidogenic enzymes and the transcription factor, GATA-binding factor 6 (GATA6) has previously been reported to increase promoter activities of *CYP11A* and *CYP17A* [38]. In the theca cells of High A4 cows, the expression of *LHCGR* (*P* = 0.01, Fig. 4B) mRNA was increased 13.1-fold and *GATA6* (*P* = 0.01, Fig. 3E) mRNA abundance was increased 30-fold. Although we report increased expression of steroidogenic enzyme gene expression in theca from High A4 cows, there was no difference (*P*≥0.19) in *HSD3B* or *CYP19A1* steady-state transcript levels in granulosa cells collected from High A4 and Low A4 dominant follicles (data not shown).

### The mRNA abundance of ZAR1 and NLRP5 genes was altered in COCs obtained from High A4 compared to Low A4 cows

**Figure 5:**
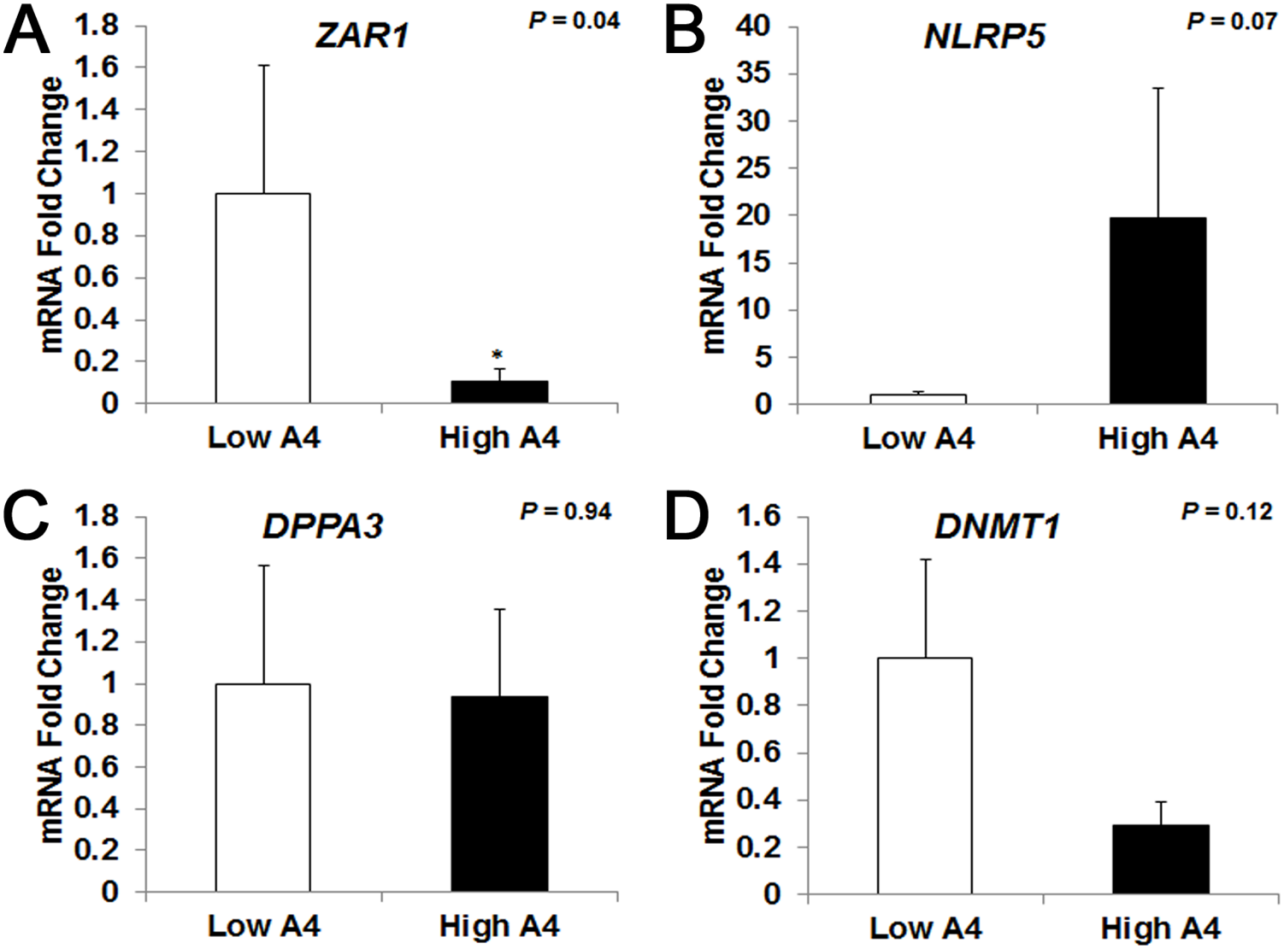
Maternal Effect Gene ZAR1 mRNA Abundance is Reduced in High A4 Compared with Low A4 Cows. Quantitative RT-PCR results for *ZAR1* (A), *NLRP5* (B), *DPPA3* (C), and *DNMT1* (D); in cumulus oocyte complexes of High A4 (black bars, n≥3) and Low A4 (white bars, n≥5) cows. The geometric mean of *GAPDH* and *RPL-15* was used as an endogenous control. Graphs represent a fold change in mRNA abundance with Low A4 set as control (1). Data for *NLRP5* and *DPPA3* were log transformed to meet normal distribution assumptions. The mean ± SEM normalized values are presented. *, *P*≤0.05.

To determine the effect of altered follicular fluid concentrations of steroid hormones on the molecular phenotype of the cumulus-oocyte complex, QPCR analyses of candidate genes were carried out. The abundance of two maternal effect genes was differentially expressed in COCs of High vs. Low A4 cows. Specifically, *ZAR1* mRNA was reduced 10-fold in High A4 (*P* = 0.04, Fig. 5A) compared with Low A4 cows. Likewise, *NLRP5* gene expression tended to be increased 19.8-fold in High A4 cows (*P* = 0.07, Fig. 5B). However, there was no difference in *DNMT1* (*P* = 0.12, Fig. 5C) or *DPPA3* (Fig. 5D, *P* = 0.94) mRNA abundance between High and Low A4 cows. Despite these differences in maternal effect gene transcripts, the mRNA levels of miRNA processing genes in the COCs were not different between the two cow populations. Specifically, there was no difference in expression of (DiGeorge syndrome critical region 8 (*DGCR8*), *RNASEN*, exportin 5 (*XPO5)*, *DICER*, or *EIF2C*2 (Fig. S2) based on A4 classification.

### Theca:Granulosa cell weight ratio, follicular fluid volume, and progression from preantal to antral follicle stages in ovarian cortex were altered in High A4 cows

**Figure 6:**
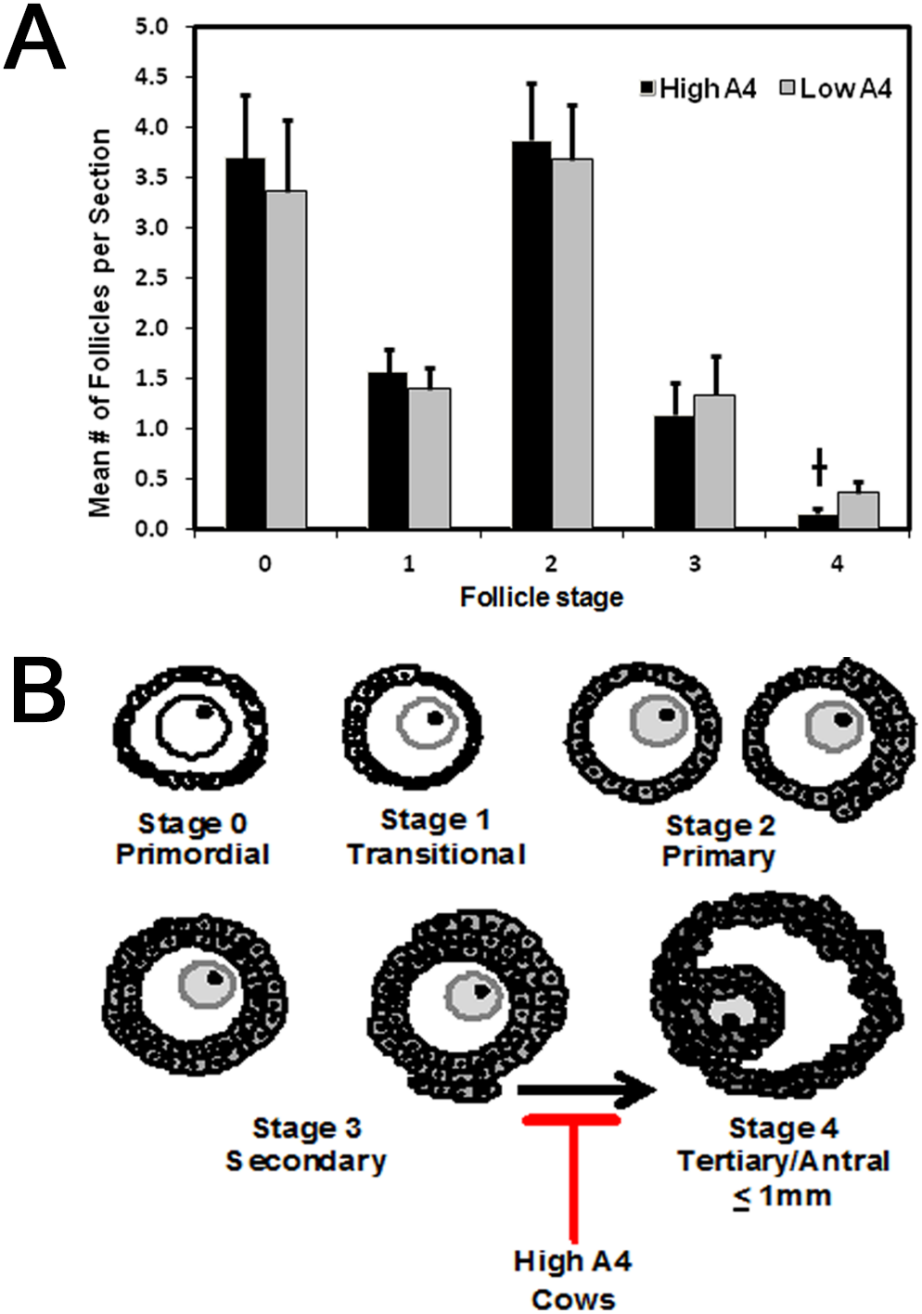
High A4 Cows Tend to have Reduced Numbers of Tertiary Follicles per Section. Follicles were classified and staged based on morphology (B) and counted on a per section basis to determine the effect of cow A4 classification on follicle growth and development. There was no difference in the number of primordial to secondary follicles (stage 0–3) between Low or High A4 cows (A). However, Low A4 cows tended to have a greater number of tertiary follicles (stage 4) compared with High A4 cows (A). ^†^, *P* = 0.06.

Given the differences identified in the dominant follicle of High A4 and Low A4 cows, additional phenotypes were measured in the ovariectomized cows and are summarized in Table 1. There was no difference (*P* = 0.57) in age for cows classified Low A4 compared with High A4 (4.31 vs. 4.75±0.61 years). After removal, ovaries were measured (length and width) and area calculated to determine the effect of A4 classification on ovarian size. There were no differences (*P*≥0.19) in follicle diameter, ovarian weight, theca or granulosa cell weight, or antral follicle count (AFC) based on A4 classification. However, the ratio of theca cells to granulosa cells (based on weight) was greater (*P* = 0.05) in High A4 compared to Low A4 cows (1.83 vs. 1.20±0.30). To determine differences in folliculogenesis within the ovarian cortex of cows from the Low and High A4 cows, hemotoxylin and eosin stained sections of ovarian cortex were analyzed to determine numbers of follicles at each stage of development. There was no difference in the number of follicles classified primordial, transitional, primary, or secondary (stages 0–3, Fig. 6A) between Low and High A4 cows. However, High A4 cows tended (*P* = 0.06) to have decreased numbers of follicles per section at stage 4 which are follicles no larger than 1 mm in diameter with a distinct antrum (Fig. 6A).

### High A4 cows tend to have a decreased calving rate

Due to alterations in steroidogenic hormone concentrations and oocyte mRNA abundance, several cow production traits were examined for all Low and High A4 classified cows (aspirated and ovariectomized). High A4 cows tended (*P* = 0.07) to have a decreased calving rate (17% reduction) compared to Low A4 cows (Table 3). However, A4 classification did not impact cow age at puberty or age at first calving. Interestingly, when High A4 cows did produce a calf, the weaning weight of the calf was on average 12 kg heavier (*P* = 0.04) than calves produced by a Low A4 cow suggesting that there may be differences in utero that are affecting calf growth at later stages of development.

**Table 3:** Average age and reproductive parameters for Low and High A4 cows.

Item	Low A4^1^	High A4^2^	SEM	*P*-value
n	59	46		
Age, years	4.31	4.75	0.61	0.57
Cow age at puberty, days	327	282	23	0.17
Age at first calving, days	736	730	13	0.72
Produced calf, %	78	61	7	0.07
Calf weaning weight, kg	249	261	4	0.04

1 Dominant follicle follicular fluid A4 concentration of <20 ng/mL.

2 Dominant Follicle Follicular Fluid A4 Concentration of >40 ng/mL.

## Discussion

In the current study, we identified a population of cows within our research herd with high follicular fluid androgen concentrations in the dominant E2-active follicle. This population of High A4 cows exhibited altered follicular steroid profiles, increased theca steroidogenic enzyme mRNA abundance and reduced ZAR1 COC mRNA abundance, as well as potential arrest in follicle progression from the preantral to antral stage in the ovarian cortex. Furthermore, the High A4 cows exhibited a 17 percent reduction in calving rates (Table 3); suggesting that the altered follicular and COC phenotypes contribute to impaired fertility in this population of cows.

Androgen synthesis in the bovine ovary is dependent on the expression of *CYP11A1, CYP17A1,* and *HSD3B.* Cattle predominantly utilize the Δ^5^ steroidogenic pathway [1], [39]. Therefore, the conversion of cholesterol to pregnenolone is accomplished by CYP11A1. Pregnenolone is converted first to 17-OH pregnenolone and then DHEA by CYP17A1 followed by production of A4 by HSD3B [1]. As expected we reported increased mRNA abundance of both *CYP11A1* and *CYP17A1* in High A4 compared with Low A4 theca cells. While, we did not detect any differences in *HSD3B* mRNA abundance in granulosa or theca cells (data not shown) there may be increases in protein abundance and/or enzyme activity which promote the high accumulation of A4 in follicular fluid of the High A4 cow population. These data are consistent with studies by Nelson et al. [40], [41] which reported that when steady state abundance of *CYP11A1* and *CYP17A1* mRNA as well as the activity of CYP17A1 and HSD3B is increased in human theca cells, basal and forskolin-induced A4 and T production by these cells is also increased. Interestingly, GATA6 mRNA abundance was also increased 30-fold in theca cells from High A4 cows. Ho et al. [12] showed that GATA6 regulates the expression of both *CYP11A1* and *CYP17A1* suggesting that changes in the expression profile of this transcription factor may be linked to excess androgens in the follicular fluid of High A4 cows.

While A4 and T were dramatically different between the High and Low A4 classified cows, there was only modest, albeit significant increases in both E1 and E2 concentrations in the follicular fluid (Table 1). Furthermore, the ratios of E1:A4 and E2:T were reduced in High A4 compared to Low A4 cows. The synthesis of E1 and E2 in granulosa cells is dependent on the aromatization of A4 and T, respectively by the P450 aromatase enzyme (CYP19A1) [1]. Furthermore, Wu et al. [42] demonstrated that testosterone increases the expression of *CYP19A1* in rat granulosa cells in a dose dependent manner and in the absence of its aromatization to E2. Thus, it was our expectation that *CYP19A1* transcription and therefore mRNA abundance should be increased in the granulosa cells from High A4 cows. However, aromatase mRNA abundance in the granulosa cells of High A4 cows was (*P*≥0.19) similar to Low A4 cows (data not shown). Due to the lack of differences in CYP19 mRNA abundance, these data point to possible abnormalities in aromatase protein translation, stability, and/or localization in the granulosa cells of High A4 cows. For example, Kumar et al. [43] showed that miR-19b and miR-106a target *CYP19A1* transcripts. Thus, altered expression of these miRNAs may alter the stability and/or translation of *CYP19A1* and thereby prevent expected increases in the aromatase enzyme resulting in poor conversion of androgens to estrogens. The conversion of androgens to estrogens is a three-step enzymatic process [44] and therefore alterations in any one of these steps could also slow the kinetics of estrogen synthesis resulting in the poor conversion rates seen in the High A4 cows.

Regulation of steroidogenic enzyme expression and activity is mediated by LH and FSH in the ovary. In cows treated with the synthetic progestin Melengesterol Acetate (MGA) for 14 days which causes the development of a persistent follicles [45], LH pulse frequency and androgen production in dominant follicles is increased along with presumably LH receptor in granulosa and theca cells [46]. Theca cells from our High A4 cows synchronized with a modified co-synch protocol had greater *LHCGR* mRNA abundance suggesting inherent changes in LH pulse frequency or sensitivity to LH that may contribute to the excess androgens (A4 and T) synthesized by the follicles of High A4 cows. In cows that have persistent follicles due to MGA-induced increased LH pulse frequency; the diameter of the follicles are greater [46]. In the current study, when estrous cycles in cows were synchronized with a modified co-synch protocol to induce a follicular wave prior to ovariectomy there was a tendency for increased follicular fluid volume in High A4 cows that may be reminiscent of persistent follicles. With this protocol, cows are given GnRH to induce ovulation and eliminate large ovulatory size follicles. Exogenous progesterone is administered for seven days and at removal of progesterone, PG is injected to lyse any CLs present. In the current study, we ovariectomized these cows 36 hours after the PG injection. Thus, the follicular waves in each of these cow classifications should have been initiated at similar time frames and should be synchronized. The fact that there is a tendency for increased follicular fluid volume in the High A4 group does suggest that LH pulse frequency or sensitivity may differ in High A4 cows to stimulate this tendency for enhanced follicular fluid production.

Precursors to aldosterone and cortisone, 11-dehydroxycortisol and 11-dehydroxycorticosterone (DOC), respectively, were also elevated in follicular fluid from dominant E2-active follicles of High A4 compared to Low A4 cows. In both primates and women, mineralocorticoids can be produced within the ovary but only after an ovulatory stimulus. Specifically, prior to ovulation cortisol is present in follicular fluid but there is no detectable levels of mineralocorticoid precursors such as 11-dehydroxycortisol and DOC. In primates there are increases in progesterone, DOC, 17- alpha hydroxyprogesterone and 11-deoxycorticol but not corticosterone or aldosterone in follicular fluid as early as 3 hours after an ovulatory stimulation with hCG [47]. Fru et al. [47], propose that progesterone and 17alpha-hydroxyprogesterone is converted to DOC and 11-deoxycortisol through 21 hydroxylase (CYP21A2) made in primate granulosa cells. The current study may be the first report that demonstrates follicular fluid levels of mineralocorticoids and their precursors in the cow; suggesting that, similar to women and primates, there is an ovarian site of mineralocorticoid synthesis in the cow.

Because DOC and 11-dehydroxycortisol are elevated in follicular fluid from High A4 cows this may provide further support that LH secretion is elevated or that increased LH receptors are more sensitive to LH actions and are promoting mechanisms that normally occur after an LH surge and/or ovulation. Fru et al. [47] propose that progesterone and 17 alpha hydroxyprogesterone are converted to either DOC or 11-dehydroxycortisol in granulosa cells through CYP21A. They also suggest that this process is necessary for appropriate lutenization of somatic cells within the follicle. Thus, in the High A4 cows it is possible that partial lutenization of granulosa cells may be occurring due to altered steroid production (increased DOC and 11-dehydroxycortisol) and, as this partial lutenization occurs, aromatase activity is decreased and there is less conversion of androgens to estrogens which is contributing to the phenotype of decreased E2/T and E1/A4 in our High A4 cows.

Even though precursors to aldosterone and cortisone, DOC and 11-deoxycortisol were elevated in the follicular fluid of High A4 cows both aldosterone and cortisone were reduced by 2–3 fold. Studies in women suggest that high levels of mineralocorticoids in preovulatory follicular fluid may contribute to oocyte developmental competence [48] since higher accumulation of aldosterone is correlated to increased estrogen levels in follicles from younger women. Furthermore, the genes that increase accumulation of corticosterone, a precursor to aldosterone, also are elevated in patients that are predisposed to be more fertile. One of the genes, CYP19A1 or P450 aromatase, has been shown in mice to promote, presumably via increased E2 levels, corticosterone synthesis [49]. While there were no differences in corticosterone between our cow classifications there were significant reductions in aldosterone production which may have resulted indirectly from reduced aromatase activity. Aldosterone receptor is also present on human oocytes and is thought to directly affect oocyte development [48]; the mechanism for how this occurs is unclear but it is something we plan to evaluate in our two cow populations.

In addition to the altered steroid profiles of High A4 cows, examination of the ovarian cortex identified decreased numbers of follicles at stage 4 which was defined as follicles no larger than 1 mm in diameter with a distinct antrum (Fig. 6A). These data suggest an abnormality in the transition from the preantral to antral stage of follicle growth. This result is somewhat unexpected given recent studies which show androgen dependent increases in follicular development [50], [51]. However, these studies acutely treated granulosa cells or ovarian cortex tissue in *in vitro* systems using much lower levels of androgens compared to the intrafollicular A4 concentrations in the dominant follicle of High A4 cows. Furthermore, Orisaka et al. [52] showed that chronic exposure of preantral follicles to LH decreased FSHR expression and FSHR-mediated signaling resulting in reduced follicular growth. Based on these data we propose that chronic exposure to increased androgens and potentially increased LH contributes to the block in follicle progression from the preantral to antral stage in the High A4 cows (Fig. 6B). Despite the difference in stage 4 follicles in the ovarian cortex of High A4 cows, no differences in stage 0 (primordial follicles) or stages 1–3 (growing preantral follicles) between the 2 cow populations were detected. Using an androgenized ewe model, Forsdike et al. [53] showed a decrease in primordial and an increase in growing preantral follicles when ewes were 8 months of age. However, this difference was no longer detectable when the ewes were 20 months of age. In the current study, the average age of the cows was 4.31 years (52 months) and 4.75 years (57 months) in the Low and High A4 cows, respectively. Thus the lack of differences in the primordial and growing preantral follicles between High and Low A4 cows is also consistent with this previous study.

Previous reports suggest that ovarian derived androgen excess in women reduces oocyte quality [54], [55]. Oocytes that are subjected to increased LH pulse frequency for extended periods of time due to prolonged or persistent follicular waves are also less competent for fertilization and embryo development which has been attributed to oocyte “aging” [56]. Reduced oocyte developmental competence may also be due in part to differential expression of maternal effect genes [17] that are important in promoting survival during early embryogenesis [18]. Indeed, loss of maternal effect transcripts/proteins results in arrested development of the pre-implantation embryo [57]. In the current study, we identified a 10-fold decrease in *ZAR1* mRNA abundance in High A4 cows which has been reported to have a role in regulating RNA stability/translation in embryos [58]. Thus, altered gonadotropin support and increased androgens may contribute to the altered expression of ZAR1 mRNA abundance in our High A4 model which may be a factor resulting in the 17% reduction in calving rate.

Circulating concentrations of androstenedione in plasma did not differ between the Low or High A4 cow classifications. Thus, plasma samples cannot be utilized to determine their classification. To determine if there were differences in antral follicle count we collected data prior to and after ovariectomy on antral follicle count through ultrasound and ovarian surface counts. There were no differences in number of antral follicle counts between cow classifications. To confirm this we also analyzed plasma samples from cows and determined that circulating concentrations of AMH are also not different. Additional production traits in the two cow classification were collected which determined that in addition to reductions in calving rate, the High A4 cows also had increased weaning weight in their progeny (12 kg or 26.4 lbs) suggesting something inherent during gestation may be affecting growth of these offspring. This increase in weaning weight may also demonstrate why these cows are maintained in the herd. While the age at puberty in these cows were not different (*P*<0.17); on average the High A4 cows obtained puberty 45 days earlier than the Low A4 cows. As cows are added to our High and Low A4 classifications; age at puberty will be monitored to see if these traits become significantly different.

The bovine model is an appropriate model to understand reproductive inefficiencies in humans. Similar to humans, cows are monovular [24] and similarities in ovarian size and morphology exist between humans and cattle. Ovarian follicle size is similar between species (15 to 20 mm in diameter) and similar pathologic conditions can occur in both species [23]. While the current bovine model does not fully recapitulate PCOS in women, it has many aspects which are similar including intra ovarian androgen excess, overexpression of steroidogenic enzymes and *LHCGR* within the theca, altered maternal effect gene expression and some aspects of follicular arrest within the ovary. Furthermore, to date no naturally occurring animal model exists for studying androgen excess disorders or anovulation; which due to the increase in calf progeny weaning weight may recapitulate into an economic advantage to producers if they can continue to get these cows pregnant and obtain a calf. Thus, this cow model appears to be a promising new animal model for examining the mechanistic problems associated with intra-ovarian androgen excess and its effects on female fertility and progeny phenotypes.

## Supporting Information

Figure S1 **Estrous Synchronization Protocol Prior to Aspiration or Ovariectomy.** (A) Cows received an intramuscular injection of PG on day 0 and a second injection PG on day 14 for estrous synchronization. Following the second administration of PG dominant follicles were aspirated from 6–60 hours after stimulation. (B) Cows received an intramuscular injection of gonadotropin-releasing hormone (GnRH) on day 0 and a controlled drug release intravaginal insert (CIDR) was inserted. The CIDR was removed on day 7 and an intramuscular injection of PG was administered. Ovariectomy was performed approximately 36 after the injection of PG.
(TIF)

Figure S2 **Cumulus Oocyte Complex miRNA Processing Gene mRNA Abundance is Similar for High and Low A4 Cows.** Quantitative RT-PCR results for *DGCR8* (A), *RNASEN* (B), *XPO5* (C), *DICER* (D), *EIF2C2* (E), in cumulus oocyte complexes of High A4 (black bars, n≥3) and Low A4 (white bars, n≥2) cows. The geometric mean of *GAPDH* and *RPL-15* was used as an endogenous control. Graphs represent a fold change in mRNA abundance with Low A4 set as control (1). Data for *DICER* and *EIF2C2* were log transformed to meet normal distribution assumptions. The mean ± SEM normalized values are presented. *, *P*≤0.05.
(TIF)

Table S1 (DOCX)
