# Supplementary figures and images for: Altered Theca and Cumulus Oocyte Complex Gene Expression, Follicular Arrest and Reduced Fertility in Cows with Dominant Follicle Follicular Fluid Androgen Excess
Source: PLoS One. 2014 Oct 16;9(10):e110683. doi: 10.1371/journal.pone.0110683 (PMC4199720; doi:10.1371/journal.pone.0110683)

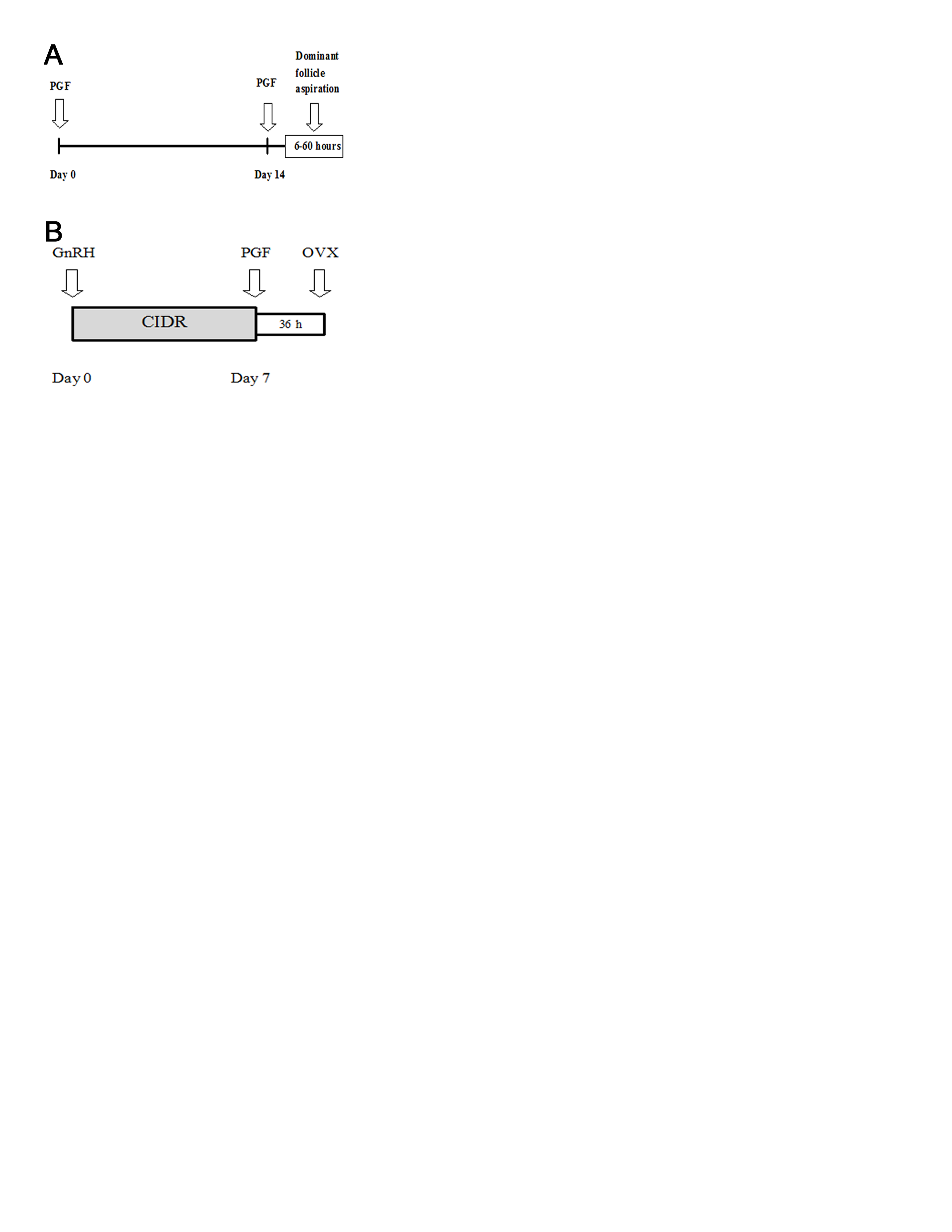

Supplement: Figure S1 — Estrous Synchronization Protocol Prior to Aspiration or Ovariectomy. (A) Cows received an intramuscular injection of PG on day 0 and a second injection PG on day 14 for estrous synchronization. Following the second administration of PG dominant follicles were aspirated from 6–60 hours after stimulation. (B) Cows received an intramuscular injection of gonadotropin-releasing hormone (GnRH) on day 0 and a controlled drug release intravaginal insert (CIDR) was inserted. The CIDR was removed on day 7 and an intramuscular injection of PG was administered. Ovariectomy was performed approximately 36 after the injection of PG. (TIF) [file pone.0110683.s001.tif]

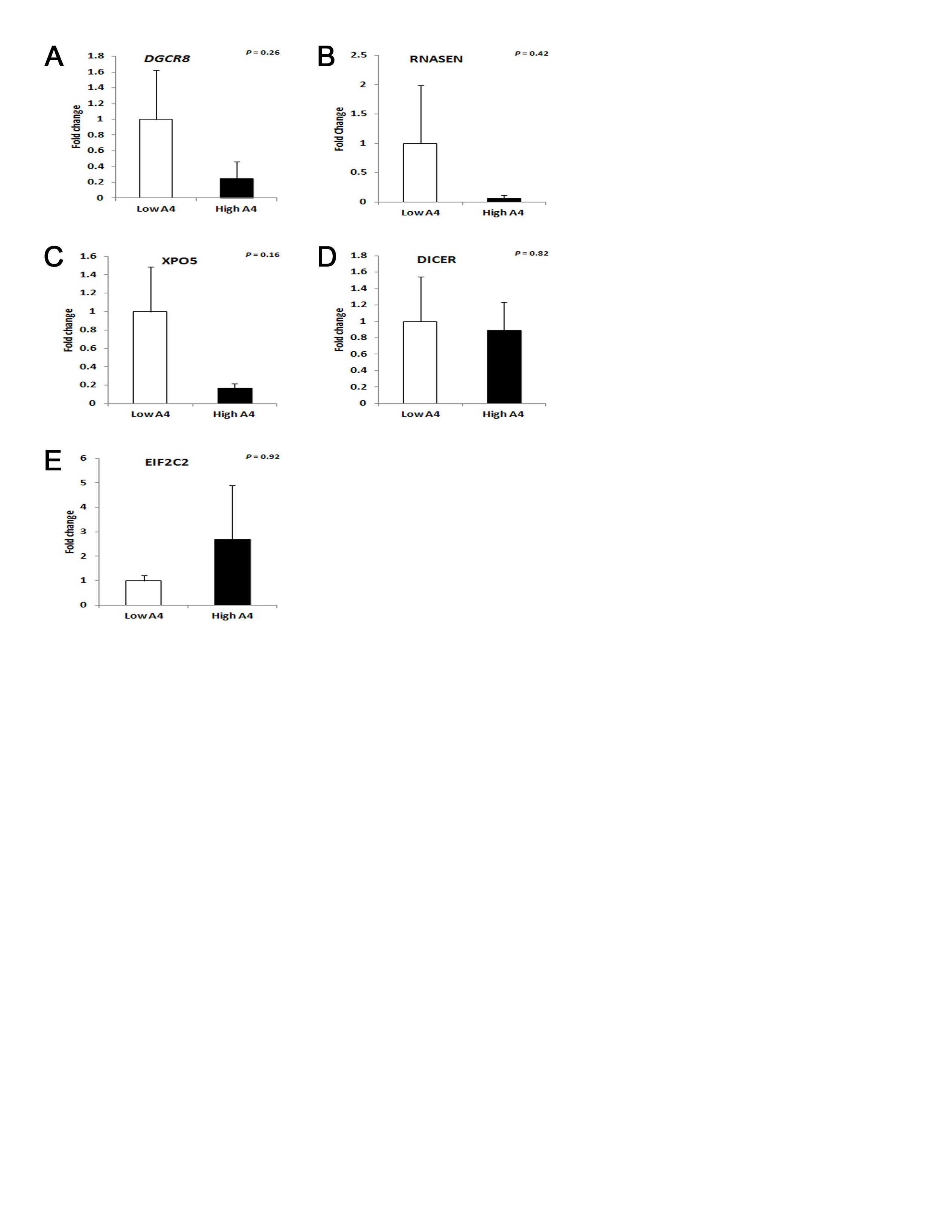

Supplement: Figure S2 — Cumulus Oocyte Complex miRNA Processing Gene mRNA Abundance is Similar for High and Low A4 Cows. Quantitative RT-PCR results for DGCR8 (A), RNASEN (B), XPO5 (C), DICER (D), EIF2C2 (E), in cumulus oocyte complexes of High A4 (black bars, n≥3) and Low A4 (white bars, n≥2) cows. The geometric mean of GAPDH and RPL-15 was used as an endogenous control. Graphs represent a fold change in mRNA abundance with Low A4 set as control (1). Data for DICER and EIF2C2 were log transformed to meet normal distribution assumptions. The mean ± SEM normalized values are presented. *, P≤0.05. (TIF) [file pone.0110683.s002.tif]
