# Supplementary material for: Altered Theca and Cumulus Oocyte Complex Gene Expression, Follicular Arrest and Reduced Fertility in Cows with Dominant Follicle Follicular Fluid Androgen Excess
Source: PLoS One. 2014 Oct 16;9(10):e110683. doi: 10.1371/journal.pone.0110683 (PMC4199720; doi:10.1371/journal.pone.0110683)
Supplement: Table S1 — (DOCX) [file pone.0110683.s003.docx]

**Table S1. Sequences for primers used to conduct quantitative PCR**

| **Gene** | **Sequence** | **Accession Number** | |  |
| --- | --- | --- | --- | --- |
| *GAPDH* | 5’-GGCGCCAAGAGGGTCAT-3’  5’-ACGCCCATCACAAACATGG-3’ | | NM_001034034 | |
| *RPL-15* | 5’-TGGAGAGTATTGCGCCTTCTC-3’ 5’-CACAAGTTCCACCACACTATTGG-3’ | | AY786141 | |
| *RPL-19* | 5’-CAGACGATACCTGAATCTAAGAAGA-3’  5’-TGAGAATCCGCTTGTTTTTGAA-3’ | | NM_001040515 | |
| *LHCGR* | 5’-CAGTCCCCCGCTTTCTCAT-3’ 5’-GTAGAGCCCCATGCAGAAGTCT-3’ | | NM_174381 | |
| *StAR* | 5’-GCGGCTCTCTCCTAGGTTCTC-3’ 5’-CCACGTCAGGGATCACTTTACTC-3’ | | XR_083945 | |
| *CYP11A1* | 5’-ACCCTFAAAGTGACTTGGTTCTTC-3’ 5’-CATGGCATAGATGGCCACTTG-3’ | | NM_176644 | |
| *CYP17A1* | 5’-TGTGGCCCCTACGCTGAT-3’  5’-CGCCAATGCTGGAGTCAAT-3’ | | XM_001251231 | |
| *GATA6* | 5’-GGAAACGAAAACCTAAGAACATAAATAAG-3’  5’-GTTGGAGTCATGGGAACAGAATTA-3’ | | XM_001253596 | |
| *DGCR8* | 5’-GCAGGAGTGAGGACAGGAAG-3’ 5’-TCGAGCACTGCATACTCCAC-3’ | | TC381697 | |
| *RNASEN* | 5’-GAGCCTCCCAAGACGAAGCT-3’ 5’-GGACTCGCATTCGGATTCAC-3’ | | XM_005221629 | |
| *XPO5* | 5’-CCTCTGGTGCTCTTCTGTCC-3’  5’-TCTCTTGCGACTCTGGGTTT-3’ | | TC363295 | |
| *DICER* | 5’-GTGGCTCTCATTTGCTGTGA-3’  5’-CGTTTTGTGGAACCTGGTCT-3’ | | TC301019 | |
| *EIF2C2* | 5’-AAGTCGGACAGGAGCAGAAA-3’ 5’-TGGCACTTCTCATCAGCTTG-3’ | | TC312997 | |
| *NLRP5* | 5’-GCTGGAGTCTCTGAGGTTGGA-3’ 5’-ATGACGATGTACAGCAAACCGT-3’ | | NM_001007814.2 | |
| *ZAR1* | 5’-ACCCTTACCGAGTGGAGGATAC-3’ 5’-TTCACTGGGCAGGAGCATCTA-3’ | | NM_001076203 | |
| *DPPA3* | 5’-CAGACTTCGCTATGCCAAAGG-3’ 5’-GCACTCTTGATCGAATCTCAGTGT-3’ | | NM_001111108.1 | |
| *DNMT1* | 5’-TCTGATGGAGAAACAAAGTCTGAAGT-3’ 5’-CCGTGGGAAATGAGATGTGAT-3’ | | NM_182651.1 | |
